# A novel quantitative body shape score for detecting association between obesity and hypertension in China

**DOI:** 10.1186/s12889-014-1334-5

**Published:** 2015-01-17

**Authors:** Shukang Wang, Yanxun Liu, Fangyu Li, Hongying Jia, Longjian Liu, Fuzhong Xue

**Affiliations:** Department of Epidemiology and Health Statistics, School of Public Health, Shandong University, PO Box 100, Jinan, 250012 China; Department of Neurology, Xuan Wu Hospital, Capital Medical University, Beijing, China; The Second Hospital of Shandong University, Jinan, China; Department of Epidemiology and Biostatistics, Drexel University School of Public Health, Philadelphia, PA USA

**Keywords:** Chinese adults, China Health and Nutrition Survey, Anthropometric indices, Obesity, Hypertension, Body shape score

## Abstract

**Background:**

Obesity is a major independent risk factor for chronic diseases such as hypertension and coronary diseases, it might not be only related to the amount of body fat but its distribution. The single body mass index (BMI), waist circumference (WC), waist to hip ratio (WHR) or waist to stature ratio (WSR) provides limited information on fat distribution, and the debate about which one is the best remained. On the other hand, the current classification of body shape is qualitative rather than quantitative, and only crudely measure fat distribution. Therefore, a synthetical index is highly desirable to quantify body shape.

**Methods:**

Based on the China Health and Nutrition Survey (CHNS) data, using Lohmäller PLSPM algorithm, six Partial Least Squares Path Models (PLSPMs) between the different obesity measurements and hypertension as well as two synthetical body shape scores (BSS1 by BMI/WC/Hip circumference, BSS2 by BMI/WC/WHR/WSR) were created. Simulation and real data analysis were conducted to assess their performance.

**Results:**

Statistical simulation showed the proposed model was stable and powerful. Totally 15,172 (6,939 male and 8,233 female) participants aged from 18 to 87 years old were included. It indicated that age, height, weight, WC, WHR, WSR, SBP, DBP, the prevalence of hypertension and obesity were significantly sex-different. BMI, WC, WHR, WSR, Hip, BSS1 and BSS2 between hypertension and normotensive group are significantly different (p < 0.05). PLSPM method illustrated the biggest path coefficients (95% confidence interval, CI) were 0.220(0.196, 0.244) for male and 0.205(0.182, 0.228) for female in model of BSS1. The area under receiver-operating characteristic curve (AUC(95% CI)) of BSS1(0.839(0.831,0.847)) was significantly larger than that of BSS2(0.834(0.825,0.842)) as well as the four single indices for female, and similar trend can be found for male.

**Conclusions:**

BSS1 was an excellent measurement for quantifying body shape and detecting the association between body shape and hypertension.

## Background

It is well known that overweight and obesity is a major independent risk factor for chronic diseases such as hypertension, coronary disease and diabetes [1,2]. Many epidemiological studies suggested a progressive increase in the prevalence of elevated blood pressure or hypertension with increasing obesity [3,4]. Several obesity measurements, including body mass index (BMI), waist circumference (WC), waist to hip ratio (WHR), and waist to stature ratio (WSR), have been proposed as markers for these chronic diseases in adults [5,6]. BMI, though most preferred [7], simply measures the mean weight under given body surface area and is affected by the amount of body fat, but unable to represent it’s distribution on human body [8]. However, one common sense is that health is not only affected by the amount of body fat but also distribution [9]. Various studies had shown that people with abdominal fat (with more weight around the waist) have higher risks of cardiovascular diseases (CVD) and other related diseases (such as hypertension, type 2 diabetes, and high cholesterol) than those with hip obesity (with more weight around the hip) [10,11]. Therefore, WC and WHR were widely used to measure abdominal fat for screening CVD and other related diseases (e.g. diabetes) in community population as well as in clinical practice [12,13]. Furthermore, WSR was recently confirmed as a better index of obesity over WC and BMI for detecting cardiometabolic risk factors [14], and it trumped BMI as screening tool for cardiometabolic risk. This indicated that body shape, which concentrates on fat distribution on human body, might be the more suitable for detecting the obesity related diseases.

However, WC, WHR or WSR, though gives a clearer indication of relative abdominal shape, still provide limited information on fat distribution. As a measurement of obesity, BMI represents a very crude index of shape, while WHR or WSR gives clearer indication of relative abdominal shape by taking account of the difference in body structure. Hence, it is possible for two man (or woman) to have vastly different BMIs but the same WHRs (or WSRs), or to have the same BMIs but vastly different WHRs or WSRs [15]. In practice, the influence of body fat distribution has been linked with body shape, which was generally broken down into four distinct types named after the fruits they resemble most [16,17], including apple (with more weight around the waist), pear (with more weight around the hip), pear-apple (with the WHR above the standard), Chilli (with normal BMI). Research shows that people with “apple” body shape face more health risks than those with “pear” body shape, and the combination of WHR and BMI is a better predictor of CVD risk and mortality than BMI alone [18]. However, this classification of body shape is qualitative rather than quantitative, and still crudely measure fat distribution on human body. Therefore, the structural equation model (SEM) between body shape and hypertension under the framework of Partial Least Squares Path Model (PLSPM) was built in this study. The synthetical index (body shape score, BSS) for quantifying body shape was constructed by the four basic measurements of BMI, WC, WHR and WSR in order to better explain the relationship between body shape and high blood pressure. Simulations are conducted to illuminate whether the proposed model is stable and efficient. Moreover, the China Health and Nutrition Survey (CHNS) data is further analyzed to assess whether the proposed model is in line with reality and to illustrate the performance of the constructed BSS to predict hypertension.

## Methods

### Study sample and measurements

CHNS data which was conducted in nine provinces of China (including Guangxi, Guizhou, Heilongjiang, Henan, Hubei, Hunan, Jiangsu, Liaoning and Shandong) from 1993 to 2012, was used to construct our PLSPM between body shape and hypertension for both statistical simulation and real data analysis. Details about the CHNS have been published elsewhere [19]. In order to use enough sample to validate the proposed model, we used most waves of CHNS study (1993, 1997, 2000, 2004, 2006 and 2009) and took the first survey result for repeated measurements of each individual. A total of 25,753 individuals aged 18 years or older were potential eligible in this study. However, 10,581 persons were excluded since they did not provide anthropometry data such as height, weight, WC, hip circumference, systolic blood pressure (SBP), diastolic blood pressure (DBP) and current use of antihypertensive medications. At last, 15,172 (6,939 male and 8,233 female) participants with 18 to 87 years old were included in the final data analysis. All participants underwent a standardized medical examination included the collection of a systolic blood pressure (SBP), diastolic blood pressure (DBP). Blood pressure (BP) was measured by trained examiners on the right arm using a mercury sphygmomanometer according to a standard protocol [20]. The three BP values were measured and the average of three readings was chosen as the BP values. Anthropometric measurements were taken after the participants had removed their shoes, heavy clothing, and belts. Height, weight, WC and hip circumference (Hip) were measured by the trained nurses. BMI was calculated as weight (kg)/height^2^ (m^2^), WC was measured at the level midway between the lower rib margin and the iliac crest while the participants breathed out gently. Hip was taken at the level of maximal gluteal protrusion. WHR and WSR were calculated as WC(cm)/Hip(cm) and WC(cm)/stature(cm) respectively. The presence of hypertension was defined by an SBP ≥ 140 mmHg, a DBP ≥ 90 mmHg or self-reported current use of antihypertensive medications [21]. All participants provided written informed consent and the study was approved by institutional review committees of the University of North Carolina at Chapel Hill and the National Institute for Nutrition and Food Safety, Chinese Center for Disease Control and Prevention.

### Model structure

**Figure 1:**
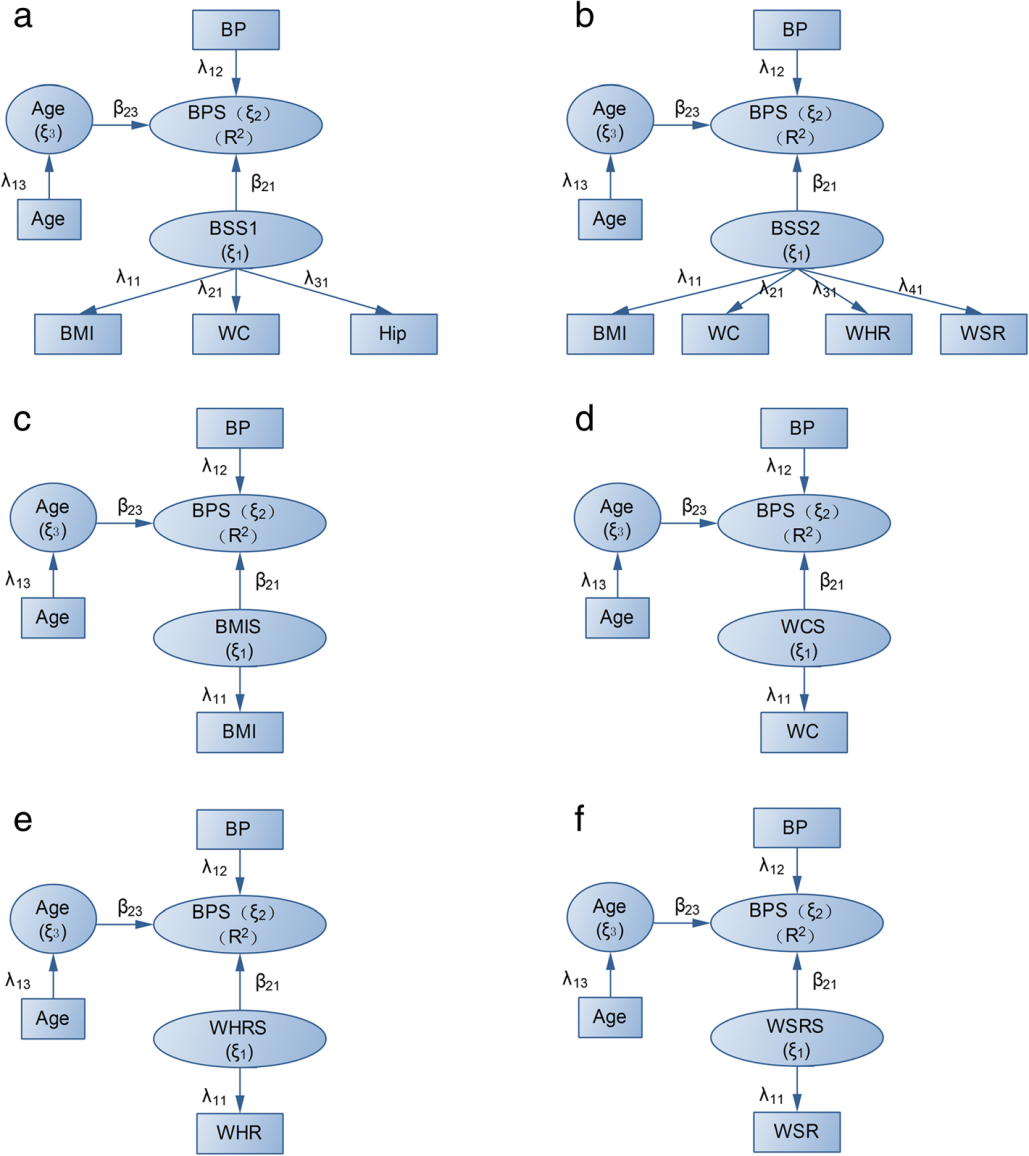
**PLSPM-based models.** The six models for detecting the association between obesity and hypertension with different manifest anthropometric measurements, with **(a)** using BMI/WC/Hip, **(b)** BMI/WC/WHR/WSR, **(c)** BMI; **(d)** WC; **(e)** WHR; **(f)** WSR.

Figure 1 showed the model structure between hypertension and body shape under framework of PLSPM using different manifest anthropometric measurements, with (a) by BMI/WC/Hip, (b) by BMI/WC/WHR/WSR, (c) by BMI; (d) by WC; (e) by WHR; (f) by WSR. Figure 1(a) defined a latent quantitative measurement of body shape score (*ξ*_1_) extracted from BMI, WC and Hip (BSS1), while BSS2 from BMI, WC, WHR and WSR (Figure 1b). The latent score of blood pressure (BPS, *ξ*_2_) was summarized by binary variable of hypertension. We exemplified Figure 1(a) to explain the variables and their relationships : (1) The variables in the squared boxes were the manifest variables actually measured such as BP, age, BMI, WC and Hip; (2) The variables in the ellipses were the latent variables such as BPS(*ξ*_2_), BSS1(*ξ*_1_) and age(*ξ*_3_)calculated through the corresponding manifest variables, (3) The latent variable BSS1(*ξ*_1_) corresponded to the manifest variables BMI, WC and Hip, while the latent variable BPS(*ξ*_2_)to BP, and (*ξ*_3_)to the single variable age. Three types of parameters were included: (1) Latent variable scores (*ξ*) as combinations of their manifest variables obtained iteratively from an ordinary least squares (OLS)-type algorithm; (2) path coefficients (*β*) between dependent (*ξ*_2_) and independent latent variable (*ξ*_1_) by OLS or partial least squares (PLS); (3) loadings (*λ*) of each block of manifest variables with its latent variables by OLS, and the arrows was from the manifest variable to the latent variable when single manifest exist, otherwise the arrow direction is opposite. In this paper, the Lohmäller PLSPM algorithm was used [22].

### Non-parametric test by bootstrap procedure

As the distribution of parameters from PLSPM is unknown, significant test of path coefficients and loadings were furnished by bootstrap procedures [23,24]. Significance of a parameter (*w*) under the null hypothesis: H_0_: w = 0 and the alternative hypothesis H_1_: w ≠ 0 was tested via a normal test in the form $\mathrm{U}=\frac{\left|\mathrm{w}-0\right|}{\mathrm{se}\left(\mathrm{w}\right)}$ (e.g., $\mathrm{U}=\frac{\left|{\beta}_{21}-0\right|}{\mathrm{se}\left({\beta}_{21}\right)}$) where *se*(w) is the bootstrapped standard error.

### Data simulation

We performed a real-data-based statistical simulation under the null hypothesis (H_0_) and alternative hypothesis (H_1_) to assess the PLSPM-based body shape statistics. The simulation was based on the indices included BMI, WC, HIP, SBP and DBP in CHNS data and steps were as follows: (1) Calculated the mean and covariance matrix of BMI, WC, HIP, and generated the multivariate normal distribution data, used the R package mvtnorm [25]; (2) identified *δ* as the range of 0.1 to 0.3 (0.10, 0.15, 0.20, 0.25, 0.30) by the standardized regression coefficient of a simple regression model $\mathrm{S}\mathrm{B}\widehat{\mathrm{P}}=0.325\mathrm{B}\mathrm{M}\mathrm{I}$ based on initial data, SBP and DBP were obtained by BMI for each given *δ*. (3) Built the PLSPMs and performed the statistical simulation: the simulation data under different sample sizes were sampled from the simulated population (500,000 individuals). Under the null hypothesis (H_0_), repeat 1,000 simulations under different sample sizes (n = 500, 1000, 1500, 2000, 2500) to assess the type I error. Under the alternative hypothesis (H_1_), for each model with different effect- *δ* (*δ* =0.10, 0.15, 0.20, 0.25, 0.30), conduct 1,000 simulation under different sample sizes to assess the statistical power.

### Real data analysis

For detecting the association between body shape and hypertension, student's t-test was firstly used to test the difference of the variables (age, SBP, DBP, height, weight, Hip, WC, BMI, WHR, WSR) between male and female, as well as the difference of BMI, WC, WHR, WSR, Hip, BSS1 and BSS2 between normotension and hypertension for male and female group respectively. χ^2^ test was used to test the prevalence of hypertension and prevalence of obesity based on BMI. Pearson correlation coefficient was then used to detect correlation between the five obesity related measurements (BMI, WC, WHR, WSR, Hip). The Lohmaller PLSPM algorithm was further used to calculate BSS. Along with the risk of obesity, body shape was classified into nine [26] (1 ~ 9) types by WHR increasing under given BMI increasing (see Table 1). F test and LSD test were finally used to detect linear relationship between BSS1 and body shape type (BST).

**Table 1:** **Nine types of human body shape defined by BMI combination with WHR**

**WHR**		**BMI(kg/m** ^**2**^ **)**					
		**<24**		**24 ~ 28**		**≥28~**	
male	<0.85	Chilli	(1)	Pear	(4)	Big pear	(7)
	0.85 ~ 0.90	Chilli pear-apple	(2)	Pear-apple	(5)	Big pear-apple	(8)
	≥0.90	Chilli apple	(3)	Apple	(6)	Big apple	(9)
female	<0.80	Chilli	(1)	Pear	(4)	Big pear	(7)
	0.80 ~ 0.85	Chilli pear-apple	(2)	Pear-apple	(5)	Big pear-apple	(8)
	≥0.85	Chilli apple	(3)	Apple	(6)	Big apple	(9)

BMI, body mass index; WHR, waist to hip ratio.

Apple shape referred to more weight around the waist (e.g. WHR greater than 0.9 in male), as BMI increases, the body shape varied from “Chilli apple”, to “Apple” and then to “Big apple”, so as the “Pear” and “Pear-apple” shape.

Based on the PLSPM of BSS1 and BSS2, the association between body shape and hypertension was obtained using path coefficients from BSS1/BSS2 to BPS. Six PLSPMs were created by defining the measurement model using BMI/WC/Hip, BMI/WC/WHR/WSR, BMI, WC, WHR, WSR as the manifest variable of body shape respectively (see Figure 1). By comparing the path coefficients (*β*_21_) in the six different models, we assessed their performances for detecting the association between body shape and hypertension. Furthermore, the area under receiver-operating characteristic (ROC) curve (AUC) was used to evaluate the performance of the six different BSS for predicting hypertension by adjusting age in the PLSPMs. The SmartPLS2.0 was used to build PLSPMs, SPSS16.0 was used to run t test, F test and χ^2^ test. ROC curve was created by the Medcalc program version 9.3.9.0 ( MedCalc software, Mariakerke, Belgium ).

## Results

### Simulation

**Figure 2:**
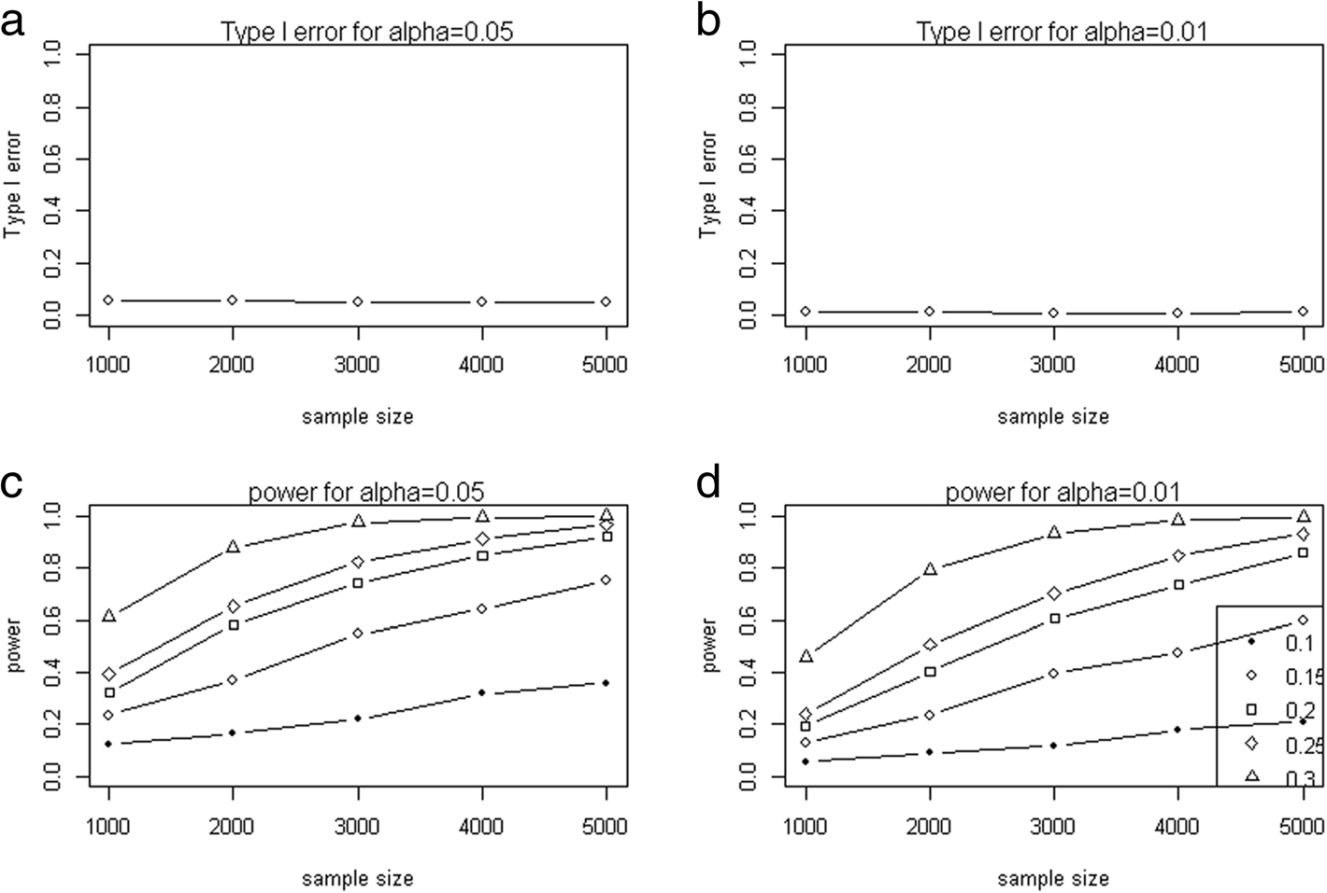
**The performance of PLSPM based model in Figure** 1 **(a) in type I error and power.** With **(a)**, **(c)** for *α* = 0.05, and **(b)**, **(d)** for *α* = 0.01.

As shown in Figure 2, the type I error rates of PLSPM model was close to nominal levels (0.05, 0.01) as a function of sample sizes (Figure 2a and b), and the power monotonically increases with sample size, effect size (δ) at *α* = 0.05 level and *α* = 0.01 (Figure 2c and d). This indicated that our PLSPM was suitable for detecting the association between body shape and hypertension.

### The real data analysis

The distribution of age, blood pressure, and obesity related variables between male and female were summarized in Table 2, and the prevalence of hypertension and obesity based on BMI are listed in Table 2. Definition of obesity based on BMI is in accordance with the standards of China [27]. It showed that age, height, weight, WC, WHR, WSR, SBP, DBP, the prevalence of hypertension and obesity were significantly different between the two groups, suggesting that PLSPM should be constructed for male and female respectively. Furthermore, the distribution of the obesity related measurements (BMI, WC, WHR, WSR, Hip, BSS1, BSS2) between hypertension and normotensive group are showed in Table 3 for male and female respectively. It demonstrated that all the variables were significantly different between hypertension and normotensive group for both male and female, suggesting that the BPS should be obtained from binary variable (hypertension = 1 *vs* normotensive = 0 ) for developing PLSPM.

**Table 2:** **Summary statistics and comparison of anthropometric measurements in different gender (mean ± s.d.)**

**Variale**	**Males(n = 6939)**	**Females(n = 8233)**
Age(years)	42.64 ± 15.44^**^	41.63 ± 15.90
SBP(mmHg)	120.13 ± 16.83^**^	115.83 ± 18.83
DBP(mmHg)	78.69 ± 10.83^**^	75.41 ± 11.37
Height(cm)	166.73 ± 6.45^**^	155.71 ± 6.17
Weight(kg)	62.38 ± 10.19^**^	54.52 ± 9.01
Hip(cm)	91.85 ± 7.93	91.69 ± 8.17
WC(cm)	79.57 ± 9.75^**^	76.54 ± 9.71
BMI(kg/m^2^)	22.38 ± 3.04	22.45 ± 3.25
WHR	0.87 ± 0.07^**^	0.83 ± 0.08
WSR	0.48 ± 0.06^**^	0.49 ± 0.06
Hypertension	24%^**^	18.0%
Obesity	5.1%^*^	5.8%

BMI, body mass index; WC, waist circumference; WHR, waist to hip ratio; WSR, waist to stature ratio; SBP, systolic blood pressure; DBP, diastolic blood pressure.

*P < 0.05, **P < 0.001: comparison between males and females (unpaired student’s t-test for numerical data, χ^2^ test for categorical data).

**Table 3:** **Sex-specific values of anthropometric indicators among normotensive and hypertensive individuals (mean ± s.d.)**

**Characteristics**	**Male**		**Female**	
	**Normotension**	**Hypertension**	**Normotension**	**Hypertension**
	**(n = 5277)**	**(n = 1662)**	**(n = 6753)**	**(n = 1480)**
BMI(kg/m^2^)	21.96 ± 2.72	23.71 ± 3.54^**^	22.05 ± 2.94	24.26 ± 3.90^**^
WC(cm)	78.01 ± 8.87	84.51 ± 10.69^**^	75.12 ± 8.87	83.01 ± 10.69^**^
WHR	0.86 ± 0.06	0.89 ± 0.07^**^	0.83 ± 0.07	0.87 ± 0.08^**^
WSR	0.47 ± 0.05	0.51 ± 0.06^**^	0.48 ± 0.06	0.54 ± 0.07^**^
Hip	90.88 ± 7.47	94.94 ± 8.56^**^	90.80 ± 7.62	95.76 ± 9.26^**^
BSS1	48.11 ± 4.45	51.29 ± 5.55^**^	48.14 ± 4.60	52.10 ± 5.86^**^
BSS2	1.09 ± 0.10	1.16 ± 0.12^**^	1.12 ± 0.11	1.22 ± 0.13^**^

BMI, body mass index; WC, waist circumference; WHR, waist to hip ratio; WSR, waist to stature ratio; Hip, hip circumference; BSS, body shape score.

^**^P < 0.001 compared to normotension in the respective group.

Table 4 showed the correlation matrix of BMI, WC, WHR, WSR, hip for male and female group. It illustrated that strong correlation between them existed, suggesting that the reflective PLSPM should be selected for defining the measurement model.

**Table 4:** **Correlation coefficient between BMI, WC, WHR, WSR and Hip**

**Variable**	**Male**				**Female**			
	**BMI**	**WC**	**WHR**	**WSR**	**BMI**	**WC**	**WHR**	**WSR**
BMI	-	0.757^**^	0.434^**^	0.751^**^	-	0.725^**^	0.315^**^	0.709^**^
WC	0.757^**^	-	0.698^**^	0.948^**^	0.725^**^	-	0.673^**^	0.950^**^
WHR	0.434^**^	0.698^**^	-	0.722^**^	0.315^**^	0.673^**^	-	0.697^**^
Hip	0.673^**^	0.786^**^	-	-	0.695^**^	0.726^**^	-	-

BMI, body mass index; WC, waist circumference; WHR, waist to hip ratio; WSR, waist to stature ratio; Hip, hip circumference.

**P < 0.001.

Table 5 showed the path coefficient from BSS to BPS in the six PLSPM models (see Figure 1) for male and female respectively. It indicated that the biggest path coefficient was in model of BSS1 → BPS, followed by BSS2, and other single index for both male and female groups, suggesting that the synthetical BSS have better performance than the single one for detecting the association between body shape and hypertension. It demonstrated that the AUC of BSS1 was significantly larger than that of BSS2 as well as the four single indices for female, and so as in males though no statistical significance can be found.

**Table 5:** **Path coefficient to BP and AUC for hypertension of anthropology indices in different PLSPM**

**Variable**	**Male**		**Female**	
	**Path coefficient (95% CI)**	**AUC (95% CI)**	**Path coefficient (95% CI)**	**AUC(95% CI)**
BMI	0.208(0.184,0.232)	0.767 (0.757, 0.777)	0.187(0.164,0.210)	0.835*(0.827,0.843)
WC	0.215(0.191,0.239)	0.767 (0.757, 0.777)	0.192(0.170,0.214)	0.834*(0.826,0.842)
WHR	0.134(0.111,0.157)	0.747*(0.736, 0.757)	0.079(0.057,0.101)	0.816*(0.808,0.825)
WSR	0.201(0.176,0.226)	0.760*(0.750, 0.770)	0.182(0.158,0.206)	0.831*(0.822,0.839)
BSS1	0.220(0.196,0.244)	0.769 (0.759, 0.779)	0.205(0.182,0.228)	0.839(0.831,0.847)
BSS2	0.217(0.194,0.240)	0.767 (0.757,0.777)	0.192(0.169,0.215)	0.834*(0.825,0.842)

BMI, body mass index; WC, waist circumference; WHR, waist to hip ratio; WSR, waist to stature ratio.

*Significant difference compared with the BSS1 (p < 0.05).

Table 6 showed the relationship between quantitative body shape score (BSS1) and qualitative body shape type (BST), suggesting that, 1) there was significant sex difference of overall body shape types ( χ^2^ = 242.55, P < 0.0001); 2) for both male and female, along with the risk of obesity, BSS1 was monotonically increasing from types 1 to 9 (Table 6), with significant differences between given two types except type 7 and type 8 (F = 1431.84, P < 0.0001 for male, F = 1738.24, P < 0.0001 for female, both with p < 0.05 according to LSD test). 3) Linear regression between the BSS1 and BST had good fit for male (F = 10988.28, P < 0.0001, R^2^ = 0.61) and female (F = 12860.00, P < 0.0001, R^2^ = 0.61), indicating that BSS1 was an excellent measurement for body shape.

**Table 6:** **Distribution of body shape types and characteristics of body shape score (BSS1, mean ± s.d.) by sex**

**Body shape types**	**Symbol**	**Male**			**Female**		
		**n**	**%**	**BSS1**	**n**	**%**	**BSS1**
Chilli	A (1)	2631	37.90	45.84 ± 3.03	2398	29.10	45.54 ± 2.95
Chilli pear-apple	B (2)	1530	22.00	47.27 ± 2.97	1688	20.50	46.85 ± 2.94
Chilli apple	C (3)	954	13.70	48.37 ± 3.41	1788	21.70	47.54 ± 3.68
Pear	D (4)	270	3.90	52.41 ± 2.88	310	3.80	51.67 ± 2.69
Pear-apple	E (5)	474	6.80	53.48 ± 2.58	550	6.70	52.88 ± 2.41
Apple	F (6)	729	10.50	54.34 ± 2.85	1018	12.40	54.05 ± 2.85
Big pear	G (7)	23	0.30	57.68 ± 3.24	26	0.30	58.09 ± 3.66
Big pear-apple	H (8)	66	1.00	58.46 ± 3.24	111	1.30	58.80 ± 3.22
Big apple	I (9)	262	3.80	60.17 ± 3.13	344	4.20	60.34 ± 3.55
Total	-	6939	100.00	48.87 ± 4.93	8233	100.00	48.85 ± 5.08

## Discussions

Obesity is an independent risk factor for hypertension, and the relationship between them has been confirmed in some studies. However, hypertension might not be only affected by the amount of body fat but also distribution. Traditionally, BMI is frequently used for the assessment of obesity in a clinical setting, but it just reflected the amount of body fat rather than their distribution, which is more likely to be associated with health-related risks. To overcome this limitation of BMI, various indices have been proposed for measuring obesity, and were used to detect the association between obesity and hypertension. Generally, the four anthropometric markers (BMI, WC, WHR and WSR) have all been proved to be associated with hypertension [28,29]. Although they were reported to be associated with hypertension, the debate about which one is the best remained. Among the obesity related indices of BMI, WC, WHR and WSR, various studies showed that WC was the best predictor of hypertension not only by cross-sectional study [30] but cohort study [31], while other studies suggested that WSR should be the best predictor [32-34], and they might depend on gender [35,36] in different population. In addition, WSR was confirmed with similar predictive power to WHR for hypertension [37]. However, several studies [38-40] indicated that BMI was not less powerful than WC, WHR or WSR for predicting hypertension. These inconsistent results might due to their single index rather than using the combined information of the four obesity measurements. Actually, some studies recommended the combination of WHR and BMI [41,42], and the combination of BMI and WC [43], to overcome the shortage of the single index. These combination methods not only embody the amount of body fat but also their distribution, which hints different types of body shape. For simplicity, four basic types of body shape (Chilli, pear, apple and pear-apple) have been proposed, and the research showed that people with “apple-shaped” bodies face more health risks than those with “pear-shaped”, and the combination of WHR and BMI is a better predictor of CVD risk and mortality than BMI alone [44,45]. However, this classification of body shape is qualitative rather than quantitative, and still crudely to measure fat distribution on human body, and the quantitative body shape index is highly desirable. We thus proposed two synthetical scores (BSS1 and BSS2) to quantify body shape using PLSPM method, BSS1 is extracted from BMI, WC and Hip, while BSS2 from BMI, WC, WHR and WSR, and both of them were further used to detect the association between obesity and hypertension. Statistical simulation showed that the proposed model was stable (Figure 2a and b), and powerful (Figure 2c and d) for detecting the association between body shape and hypertension. Further real data analysis using CHNS database illustrated that BSS1 had the better performance than BSS2 as well as the single index (BMI, WC, WHR and WSR) for detecting the association between obesity and hypertension (Table 5), and for predicting hypertension (Table 5). In addition, along with the risk of body shape, BSS1 was monotonically increasing and the linear relationship between them had been proven (Table 6). In conclusion, BSS1 was an excellent measurement for quantifying body shape and for detecting the association between body shape and hypertension.

Body shape is linked with multiple anthropology indices (BMI, WC, WHR, WSR and Hip) rather than the single one. For quantifying human body shape, the SEM method should be used to extract latent synthetical body shape score from the above manifest anthropology indices. However, as the stronger multicollinearity between the anthropology indices (BMI, WC, WHR, WSR and Hip), the ordinary linear SEM usually loses power for detecting the association between body shape score and hypertension. We, therefore, adopted PLSPM method to build the SEM model for extracting BSS, and further detecting its association with hypertension. Theoretically, compared to SEM, PLSPM is robust to multicollinearity commonly encountered in anthropometric indices data (such as high correlation between BMI, WC, WHR, WSR and Hip). It is a“soft modeling” approach requiring very few distributional assumptions, variables can be numerical, ordinal or nominal, and no need for normality assumptions, while covariance-based SEM is a “hard modeling” with heavy distribution assumptions. In addition, compared with the other methods such as logistic regression, support vector machine (SVM), decision trees and naïve Baye, traditional multivariate logistic regression may fail to deal with the multiple independent variables with multicollinearity. On the other hand, although Support vector machine (SVM), decision trees and naïve Bayes can be used for discriminant analysis and may not be affected heavily by multicollinearity, it seems unable for them to synthesize the manifest variables to get a latent score. Thus, PLSPM was preferred to construct the latent variable of body shape. In this study, the proposed synthetical BSS was only based on the cross-sectional survey data in CHNS study, further work should be conducted to evaluate its performance in the cohort study. On the other hand, it has become more and more clinically important to assess body shape based on the pattern of body fat distribution and redistribution, rather than to assess obesity simply based on an increased amount of body fat. The proposed BSS1, though just having a little higher AUC values, was still significantly better than other indices, indicating that BSS1 can indeed have superior performance. Actually, just like many previous large sample studies, the difference in AUC value is usually small when comparing different indices, and it seems that, to some extent, little increase cannot be unnecessary. On the other hand, the calculation of proposed BSS is not quite complex, and it is not quite difficult to use in the public health and clinical practice. In addition, this study also provides new insights to construct the better obesity index. Several recent studies proposed three-dimensional body scan and confirmed the relationships of three-dimensional body scanning results to hypertension [44]. Further, quantitative BSS based on the measurements of three-dimensional body scans should be developed for measuring the body shape and for detecting the association between obesity and hypertension accurately.

## Conclusions

In conclusion, BSS1 was an excellent measurement for quantifying body shape and detecting the association between body shape and hypertension.
